# Application of remote sensing technology to estimate productivity and assess phylogenetic heritability

**DOI:** 10.1002/aps3.11401

**Published:** 2020-11-29

**Authors:** C. Lane Scher, Nisa Karimi, Mary‐Claire Glasenhardt, Ashley Tuffin, Charles H. Cannon, Bryant C. Scharenbroch, Andrew L. Hipp

**Affiliations:** ^1^ Nicholas School of the Environment Duke University Durham North Carolina 27708 USA; ^2^ Center for Tree Science The Morton Arboretum Lisle Illinois 60532 USA; ^3^ Edge Hill University Ormskirk L39 4QP United Kingdom; ^4^ College of Natural Resources University of Wisconsin–Stevens Point Stevens Point Wisconsin 54481 USA; ^5^ The Field Museum Chicago Illinois 60605 USA

**Keywords:** biomass, phylogenetic diversity, phylogeny, prairie, productivity, remote sensing

## Abstract

**PREMISE:**

Measuring plant productivity is critical to understanding complex community interactions. Many traditional methods for estimating productivity, such as direct measurements of biomass and cover, are resource intensive, and remote sensing techniques are emerging as viable alternatives.

**METHODS:**

We explore drone‐based remote sensing tools to estimate productivity in a tallgrass prairie restoration experiment and evaluate their ability to predict direct measures of productivity. We apply these various productivity measures to trace the evolution of plant productivity and the traits underlying it.

**RESULTS:**

The correlation between remote sensing data and direct measurements of productivity varies depending on vegetation diversity, but the volume of vegetation estimated from drone‐based photogrammetry is among the best predictors of biomass and cover regardless of community composition. The commonly used normalized difference vegetation index (NDVI) is a less accurate predictor of biomass and cover than other equally accessible vegetation indices. We found that the traits most strongly correlated with productivity have lower phylogenetic signal, reflecting the fact that high productivity is convergent across the phylogeny of prairie species. This history of trait convergence connects phylogenetic diversity to plant community assembly and succession.

**DISCUSSION:**

Our study demonstrates (1) the importance of considering phylogenetic diversity when setting management goals in a threatened North American grassland ecosystem and (2) the utility of remote sensing as a complement to ground measurements of grassland productivity for both applied and fundamental questions.

Plant productivity shapes how species compete and respond to stressors (Mahaut et al., 2020) and consequently how species are distributed in space, thereby shaping the assembly of plant communities. Productivity is traditionally estimated directly by measuring aboveground biomass and cover, but remote sensing is emerging as a less time‐ and resource‐intensive tool (Heinsch et al., 2006; Yu et al., 2018). Estimating vegetation cover requires relatively little time and resources but considers growth in only two dimensions, ignoring vegetation height and density. Biomass estimates more directly measure total plant growth; however, collecting, drying, and weighing biomass are both resource intensive and destructive, limiting the practicality of biomass studies at large spatial and temporal scales.

Recent developments in remote multispectral imagery and vegetation structure mapping have improved our ability to estimate plant productivity (Cerasoli et al., 2018; Fischer et al., 2019). Multispectral vegetation indices (VIs) are a collection of ratios and transformations of light reflectance intensities detected in certain spectral bands. They are based on the light that is reflected by vegetation, which is influenced by leaf health, density, and photosynthetic activity (Xue and Su, 2017); thus, VIs have emerged as an important tool for estimating plant community productivity (Wang et al., 2010; Cavender‐Bares et al., 2017). Many VIs use the ratio of red to near‐infrared reflectance because of the correlation between the contrast in the absorption of these bands and leaf health (Myneni et al., 1995). The normalized difference vegetation index (NDVI; Tucker, 1979) is the most commonly used VI. NDVI is based on the ratio of red and near infrared reflectance and is often applied in ecological studies (Pettorelli et al., 2005), where it has been shown to be correlated with productivity in trees (Wang et al., 2004) and biomass in prairie (Wang et al., 2016) and tundra habitats (Goswami et al., 2015). Furthermore, variation in reflectance across spectra may be phylogenetically conserved (Cavender‐Bares et al., 2016, 2017), suggesting that multispectral analyses can potentially be used to remotely estimate the community phylogenetic composition and diversity.

Remote sensing techniques can also estimate productivity by providing data to model the physical structure of vegetation. Photogrammetry uses overlapping photographs to reconstruct 3D structures, producing models of the outermost layer of vegetation visible in the photographs (Baltsavias, 1999). It can accurately capture the structure of large and complex vegetation forms, both as individuals (Scher et al., 2019) and as blocks of vegetation. Light detection and ranging (LiDAR) yields a more nuanced view of the layers of vegetation that comprise a community or individual plant (Baltsavias, 1999; Dubayah and Drake, 2000), enabling the estimation of both vegetation volume and density. While LiDAR provides more detail than photogrammetry, both techniques can accurately estimate biomass from volume over small areas (Wallace et al., 2017). Furthermore, photogrammetry requires only red‐green‐blue (RGB) photographs, whereas LiDAR requires more expensive equipment (Solazzo et al., 2018).

High‐resolution satellite imagery remains prohibitively expensive for many ecology applications (Wang et al., 2010). By contrast, the cost of quality drone imagery is decreasing rapidly, making it an exciting tool for the investigation of individual plants and local landscapes (Cannon et al., 2018). Precision agriculture regularly uses high‐resolution drone‐based imagery (Zhang and Kovacs, 2012), and the decreasing costs of drone imagery are making this technology equally accessible to ecologists.

In this study, we first investigate how high‐resolution (<1.5 cm) drone‐based multispectral imagery and volume estimates correlate with field‐based estimates of grassland productivity. We compare four productivity proxies: two measured from field observations (aboveground biomass and percent vegetation cover) and two measured via drone‐based photography (individual light spectra and VIs [Table 1] and photogrammetry‐derived vegetation volume). We then use the productivity proxies to illuminate how the evolution of functional traits that influence plant productivity and competitive interactions shape the phylogenetic distribution of grassland productivity. Our study demonstrates the utility of remote sensing to address fine‐scale ecological questions efficiently and economically. Furthermore, it advances our understanding of how the evolution of plant traits and lineage diversity shape grassland ecosystem processes.

**TABLE 1:** Spectral bands and vegetation indices used in analyses.

Index	Name	Reference	Wavelength range or formula
GRE	Green		530–570 nm
RED	Red		640–680 nm
REG	Red edge		730–740 nm
NIR	Near infrared		770–810 nm
NDVI	Normalized difference vegetation index	Tucker (1979)	(NIR − R)/(NIR + R)
GNDVI	Green normalized difference vegetation index	Gitelson et al. (1996)	(NIR − G)/(NIR + G)
GDVI^2^	Generalized difference vegetation index	Wu (2014)	(NIR^2^ − R^2^)/(NIR^2^ + R^2^)

## METHODS

### Site

**FIGURE 1:**
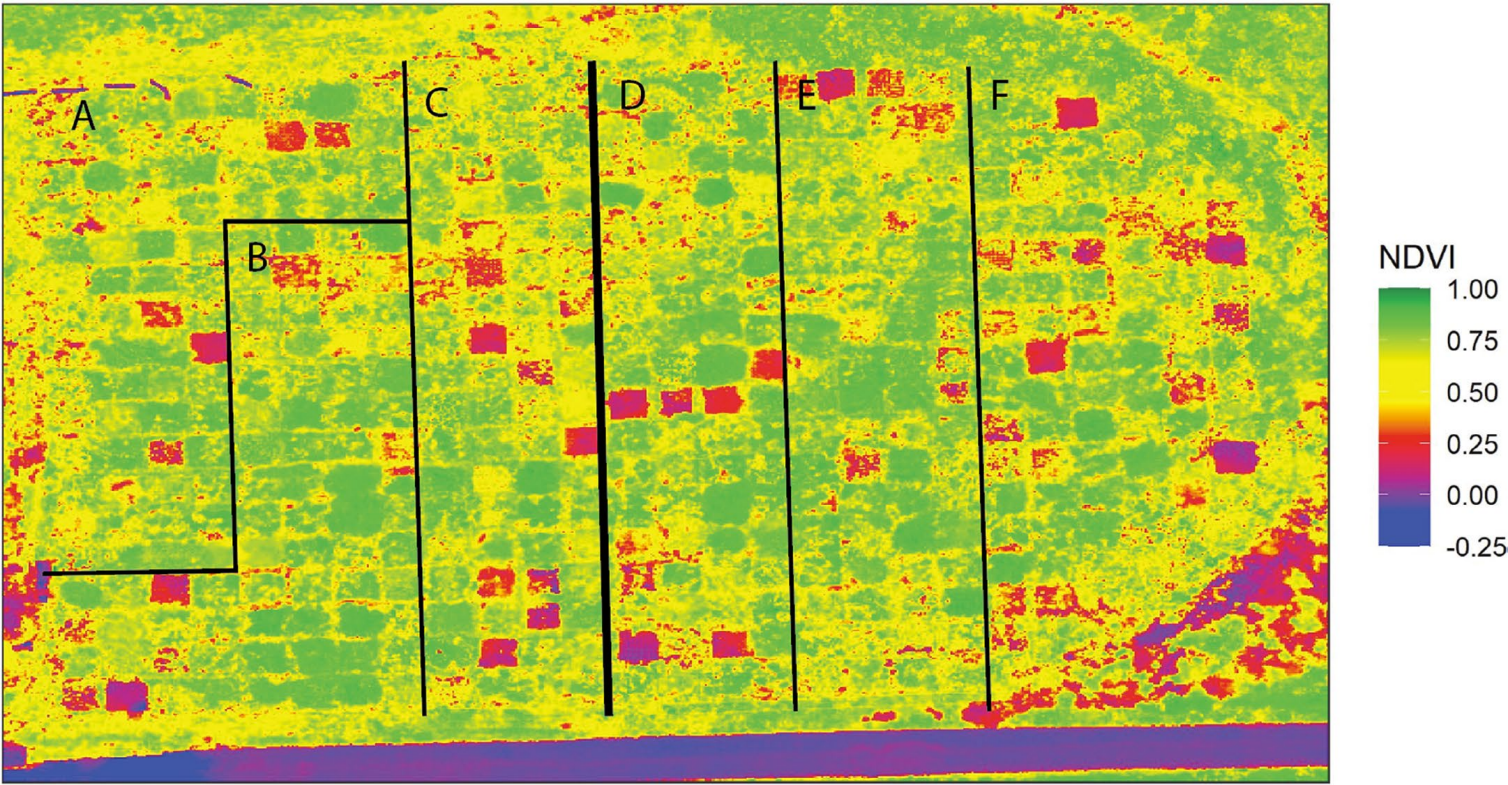
An NDVI map of the experimental prairie with blocks labeled. The thin lines mark blocks, and the thick line separates the superblocks.

Our study system is a tallgrass prairie restoration experiment located at The Morton Arboretum in northeastern Illinois, USA. The soils (Markham series, fine, illitic, mesic, mollic oxyaquic Hapludalfs) on the site are deep and moderately well drained, forming in a thin layer of silt loam loess overlying silty clay loam till (USDA‐NRCS, 2019). Our experiment manipulates phylogenetic and trait diversity in mixed species plots drawn from 127 tallgrass prairie plants, all of which essentially reached maximum productivity at the time the biomass and remote sensing data were collected (Hipp et al., 2018). The site is divided into 437 4‐m^2^ plots, which were partitioned based on measurements of their soil traits into two superblocks (west and east), each containing three blocks (A–C in the west superblock, D–F in the east superblock; Fig. 1). Of the 437 plots, our analysis used 326 plots of two types, monoculture and multispecies, all planted from plugs in August–September 2016, with follow‐up planting of failed or initially unavailable plants in summer 2017. For each of the 127 species used, we planted one monoculture plot in each superblock, for a total of 254 monoculture plots. Each multispecies plot was planted with one of 36 combinations of 15‐species mixes, and the 15‐species planting order was randomized within each quarter plot. Multispecies plots were also duplicated in both superblocks for a total of 72 multispecies plots. Complete details of the experimental design are described by Hipp et al. (2018).

### Soil analysis

We measured the A horizon in each plot and interpolated the gravimetric soil moisture, loss on ignition soil organic matter, pH, electrical conductivity, wet aggregate stability, and phosphorus from measurements of a subset of the plots. We used a principal component analysis to identify groups of plots that differed in these soil characteristics and thus partitioned the site into blocks. See Appendix S1 for details.

### Photo capture and orthomosaic construction

A DJI Phantom 4 drone (Shenzhen DJI Sciences and Technologies Ltd., Shenzhen, China) carrying a Parrot Sequoia (Parrot Drones SAS, Paris, France) was flown over the experimental prairie on 1 August 2017, during the peak of its first full growing season following establishment. The drone captured 598 RGB photos and single‐band images of the red, green, near‐infrared, and red edge bands. For details, see Appendix S1.

Seven orthomosaics were produced by merging 598 photos of the site into a single image; each orthomosaic shows one of the three VIs measured (Table 1) or one of the four wavelength bands (Table 1, Appendix S2). Values of each VI range from −1 to 1, with high values representing dense vegetation and values close to and below 0 representing non‐vegetative surfaces. Each VI, however, captures differences in vegetation density at varying levels; for example, NDVI saturates at high levels of chlorophyll concentration, and therefore does not capture variation in high vegetation densities. The green normalized difference vegetation index (GNDVI) uses green reflectance instead of red and has a larger dynamic range at high vegetation densities (Gitelson et al., 1996); GDVI^2^, a form of the generalized difference vegetation index (GDVI), is derived from NDVI and increases the dynamic range at low vegetation densities (Wu, 2014). The orthomosaics were exported from Agisoft Photoscan (Agisoft, St. Petersburg, Russia) in the tagged image file format (TIFF).

### Biomass

Vegetative material was collected between 9 October and 20 November 2017, before the first major frost, to avoid leaf disintegration. In the multispecies plots, all vegetative material in the southwest quadrant was collected. In the monoculture plots, material was collected only from the central 0.25 m^2^ of the southwest quadrant. All individuals of the planted species rooted inside the given area were clipped at ground level. The vegetation was dried in paper bags at 50°C for at least 48 h and weighed immediately after removal from the oven. We calculated the biomass of each species and the total biomass for the multispecies plots, which was converted to a dry weight of grams per square meter.

Vegetation collected early in the season from some multispecies plots in blocks A, B, C, and F was not dried sufficiently before its biomass was measured. Biomass measurements in these plots were biased upward in the preliminary analyses, so multispecies plots that were affected are excluded from all biomass analyses.

### Vegetation cover

Vegetation cover was evaluated using two methods: cover of only planted species (“planted cover”) and cover of all species (“total cover”). The planted cover was estimated by observers in the field (29 August 2017 to 1 September 2017) for all plots, using the midpoints of 14 cover classes (producing 0%, 0.5%, 3%, 7.5%, 15%, 25%, 35%, 45%, 55%, 65%, 75%, 85%, 92.5%, 97%, and 99.5% classes). Subsequently, two different observers estimated the total cover by examining the RGB orthomosaic. Each independently assessed the total percentage of each plot covered by vegetation, and their estimates were then averaged.

### Spectral imagery

All spectral band and VI orthomosaics (Table 1) were processed as rasters using the raster package (Hijmans and van Etten, 2012) in R (R Core Team, 2019). The outer 0.25 m of each plot was removed to avoid counting turf species encroaching from the paths, which were also excluded from the manual field‐based productivity estimates. For each of the seven rasters, all pixels within the resulting 1.5 × 1.5‐m squares were averaged. These values represent all vegetation in the plot at the time when the aerial images were taken, but because only the biomass of the planted species was measured after being collected later in the growing season, we used a correction factor to adjust the orthomosaics for a more consistent comparison. The adjustment was used to correct for weeds that were present in the drone images but whose biomass was not measured, as well as growth that occurred between the photo capture and biomass measurement. For details on the correction, see Appendix S1. This procedure produced 14 spectral values for each plot, representing the raw and corrected values from each of the seven rasters.

### Estimation of vegetation volume

The vegetation volume of each plot was estimated using a vegetation height model (VHM) produced by subtracting a digital terrain model (DTM) from a digital surface model (DSM). The DSM and DTM were built in Agisoft Photoscan using all points in the dense cloud and only ground‐level points, respectively. The DSM does not distinguish vegetation height from changes in ground level, so plants on higher ground appear taller, whereas the DTM interpolates the ground level below the vegetation from ground‐level points. Subtracting the DTM from the DSM produced a VHM containing the height of vegetation after correcting for ground level. We then calculated the vegetation volume as the average height of the plot multiplied by the length and width of the plot. To minimize the effects of very slight variations in plot size, the resulting volume was then standardized to a 1.5 × 1.5‐m plot. Standardizing in this way has the effect of transforming volume estimates into average vegetation heights, as estimated in the VHM. In either case, the estimate is fundamentally different from cover estimates (which estimate the area covered by a shadow cast by the vegetation) or the VIs (based on reflectance of discrete spectra).

### Predicting productivity using models

We used a linear regression to estimate how well the remote sensing metrics predict biomass and cover using base R (R Core Team, 2019) and the lme4 package (Bates et al., 2015). We also use the MuMIn (Barton, 2009), lmer4Test (Kuznetsova et al., 2017), and qpcR (Spiess, 2018) packages to evaluate the models. Each model predicting biomass uses covariates from three groups: (1) a VI or single band, (2) total or planted cover, and (3) photogrammetry‐modeled volume (hereafter, “volume”). Because the remote sensing metrics were calculated based on all the vegetation in each plot, we modeled total cover, not planted cover, using (1) a VI or single band and (2) volume. Covariates and responses were scaled to unit variance and a mean of zero prior to model fitting. Each combination of variables was run as a multiple linear regression and a mixed‐effects model using block as a random effect. All regressions were fitted using all plots, only monoculture plots, and only multispecies plots. For the multispecies plots, we modeled total biomass and cover, not partitioned by species. While there is an expected collinearity between VIs, cover, and volume, all are maintained as covariates in the models to estimate partial correlation coefficients. Although a case can be made for eliminating predictors that covary to a great extent, the retention of the relatively small number of covariates in this study is key to assessing whether there is variance in the biomass left to explain after the other potential predictors have been factored out (Legendre and Legendre, 1998).

To test whether the presence of non‐photosynthetic flowers that could alter VI values (Shen et al., 2010) affects the model fit, linear regressions were also conducted using only plots that did not have open flowers at the time of photo capture. We ran models on all nonflowering plots and on only nonflowering monoculture plots; there were only three nonflowering multispecies plots, so we did not run separate models on them.

We used *R* ^2^ to evaluate the model fit and Akaike’s information criterion (AIC) to estimate the predictive value of the models (Sober, 2002). The AIC weight (AICw) of each model was calculated as its relative likelihood as a proportion of the summed relative likelihoods of all models evaluated (following Burnham and Anderson, 2002, 2004). We then quantified the importance of each covariate by summing the AICw of all models in which the covariate was used (Burnham and Anderson, 2002) to obtain a cumulative AICw for each covariate across all models. A high cumulative AICw for a covariate indicates that the covariate is present in models that carry higher predictive weight. Unlike the AIC, the absolute value of the cumulative AICw is meaningful: a cumulative AICw near 1.0 implies that the covariate has high predictive weight relative to the population of models evaluated. In evaluating the support for individual models, we also report ΔAIC, the difference between the AIC of the best model evaluated for that test and the model being reported. While there are no hard rules about ΔAIC values, models with ΔAIC < 2 are generally considered to be practically indistinguishable in evidentiary support from the best model considered and thus to contribute significantly to our understanding of variance in the system we are studying, while those with ΔAIC > 10 have “essentially no support” (Burnham and Anderson, 2004).

### Trait data

Quantitative and qualitative trait data for each species were gathered from published sources (Iverson et al., 1999; Amatangelo et al., 2014; Sonnier et al., 2014; Ash et al., 2015; Hipp et al., 2018; Clark et al., 2019) or collected as described in Appendix S1, using the species classification described by Bai et al. (2012). Nitrogen fixation and root types (adventitious, primary, bulb, corm, fibrous, rhizome, stolon, and tuber) were coded as binary traits. Quantitative traits included the genome size, petiole length, leaf length, leaf width, leaf thickness, vegetative height, seed mass, leaf dry‐matter content, specific leaf area, leaf nitrogen content, leaf carbon content, leaf phosphorus content, stem dry‐matter content, and circularity.

### Phylogenetic and trait analyses

**FIGURE 2:**
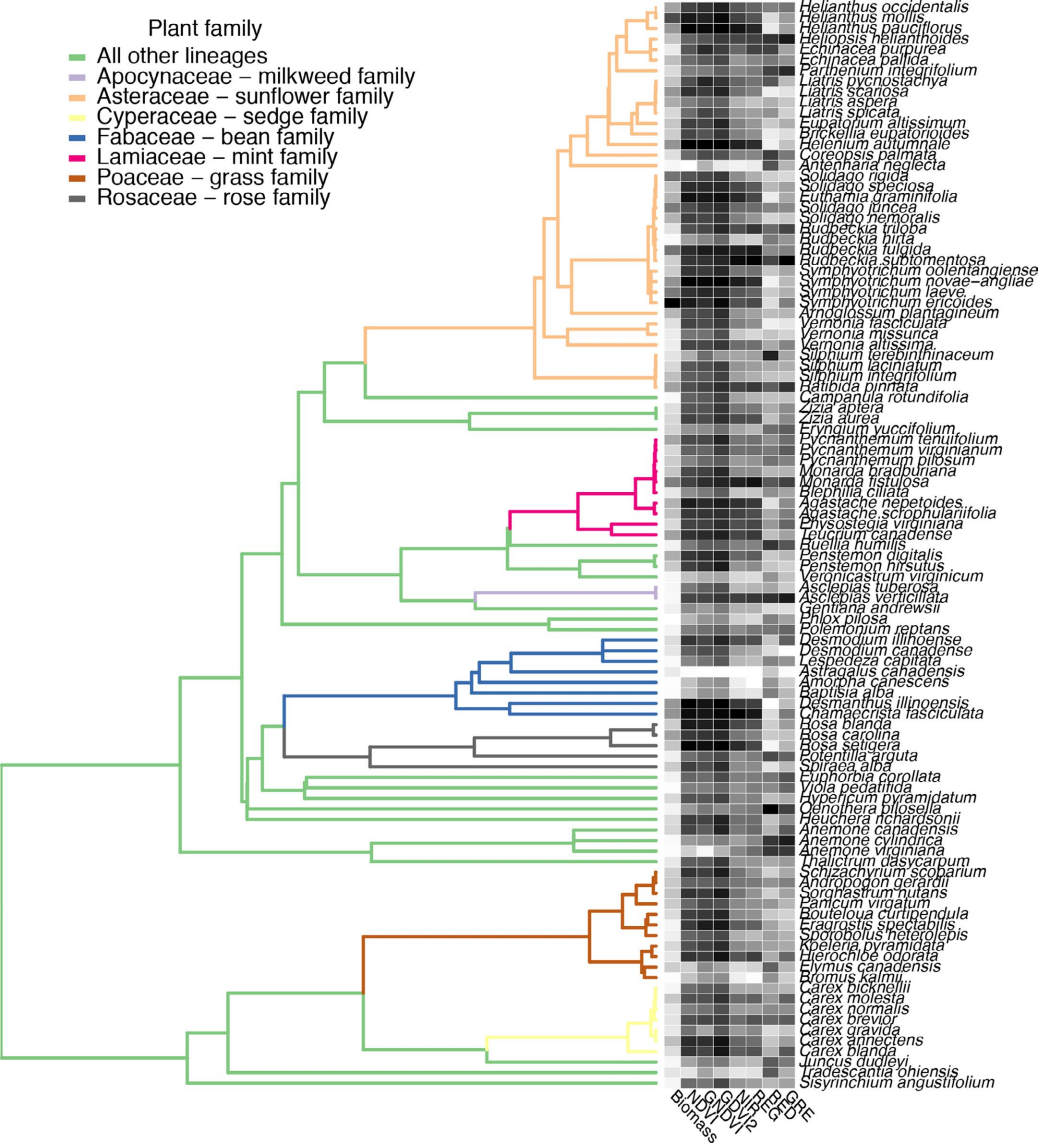
A 127‐species phylogeny with corresponding biomass measurements and spectra mapped. Trait and productivity metrics are all rescaled from 0 (white) to 1 (black); raw measurements can be found in the data sets provided in GitHub (https://github.com/lanescher/prairie-remote-sensing-2020/tree/master/DATA). Colored branches indicate dominant plant families. GRE, green; RED, red; REG, red edge; NIR, near infrared; NDVI, normalized difference vegetation index; GNDVI, green normalized difference vegetation index; GDVI^2^, generalized difference vegetation index.

To assess how trait evolution shapes the phylogenetic structure of species‐level productivity estimates in the monoculture plots, a phylogeny synthesized from published phylogenies and taxonomic knowledge of unsampled species (a “synthesis phylogeny” sensu Li et al., 2019; Fig. 2) was developed using Zanne et al. (2014) as a framework, followed by the pruning and splicing of taxa of interest in this study, as detailed by Hipp et al. (2018). We estimated the phylogenetic signal in species’ traits, biomass, and corrected VI measures from monoculture plots (see methods above) using Blomberg’s *K* (Blomberg et al., 2003) and Pagel’s *λ* (Pagel, 1999) in geiger version 2.0.6.2 (Harmon et al., 2008). The correlation (Pearson’s *r*) of plant traits with NDVI was plotted against their correlation with biomass, and the point size was scaled by the phylogenetic heritability to explore how the phylogenetic heritability of traits correlates with their influence on productivity. We then used Mantel tests of pairwise species comparisons of NDVI and phylogenetic distance, closely mirroring analyses conducted by Schweiger et al. (2018) that assessed the plant‐level correlation between the species’ mean spectral profiles and phylogenetic distance, to evaluate whether VIs at the plot level (from our study) show comparable phylogenetic heritability to the hyperspectral data at the plant level (from Schweiger et al., 2018).

We used non‐metric multidimensional scaling (Kruskal, 1964) to visualize individual species positions in the trait and productivity space, using monoMDS in the R package vegan (Oksanen et al., 2019) on a Gower’s distance matrix estimated from seven functional traits (stem dry‐matter content, leaf dry‐matter content, leaf circularity, vegetative height, leaf carbon content, leaf length, leaf thickness) along with biomass, NDVI, and GNDVI. The productivity response and trait variables were fit to the ordination in vegan using the envfit function.

The reader may note that we did not estimate partitioned biodiversity effects (sensu Loreau and Hector, 2001). The biomass sampling size in the monocultures (0.25 m^2^) in our study did not equal the sampling size in the multispecies plots (1 m^2^), and multispecies plot samples consequently included a greater proportion of plot edges than the monocultures. As the null expectation that diversity effects will equal zero is not met in the current sample, we leave these analyses to future studies.

## RESULTS

### Soil types

The first two axes of the soil principal component analysis explained 49.2% and 19.6% of the variance, respectively (available as Supplement 3 on GitHub and Zenodo [Scher et al., 2020], see Data Availability statement). The depth of the A horizon was responsible for 48.06% of the variance observed on the first axis and 23.88% of the second axis. Soils differ significantly among blocks, primarily distinguishing block A (μ = 38.5 ± 0.6 cm [SEM]) from blocks B–F (μ = 26.2 ± 0.2 cm; Kruskal–Wallis test, *k* = 172.44, *P* = 2.19 × 10^−35^; Fig. 1). Block type was consequently retained as a random effect in biomass and cover regressions to account for differences in soil.

### Biomass

**FIGURE 3:**
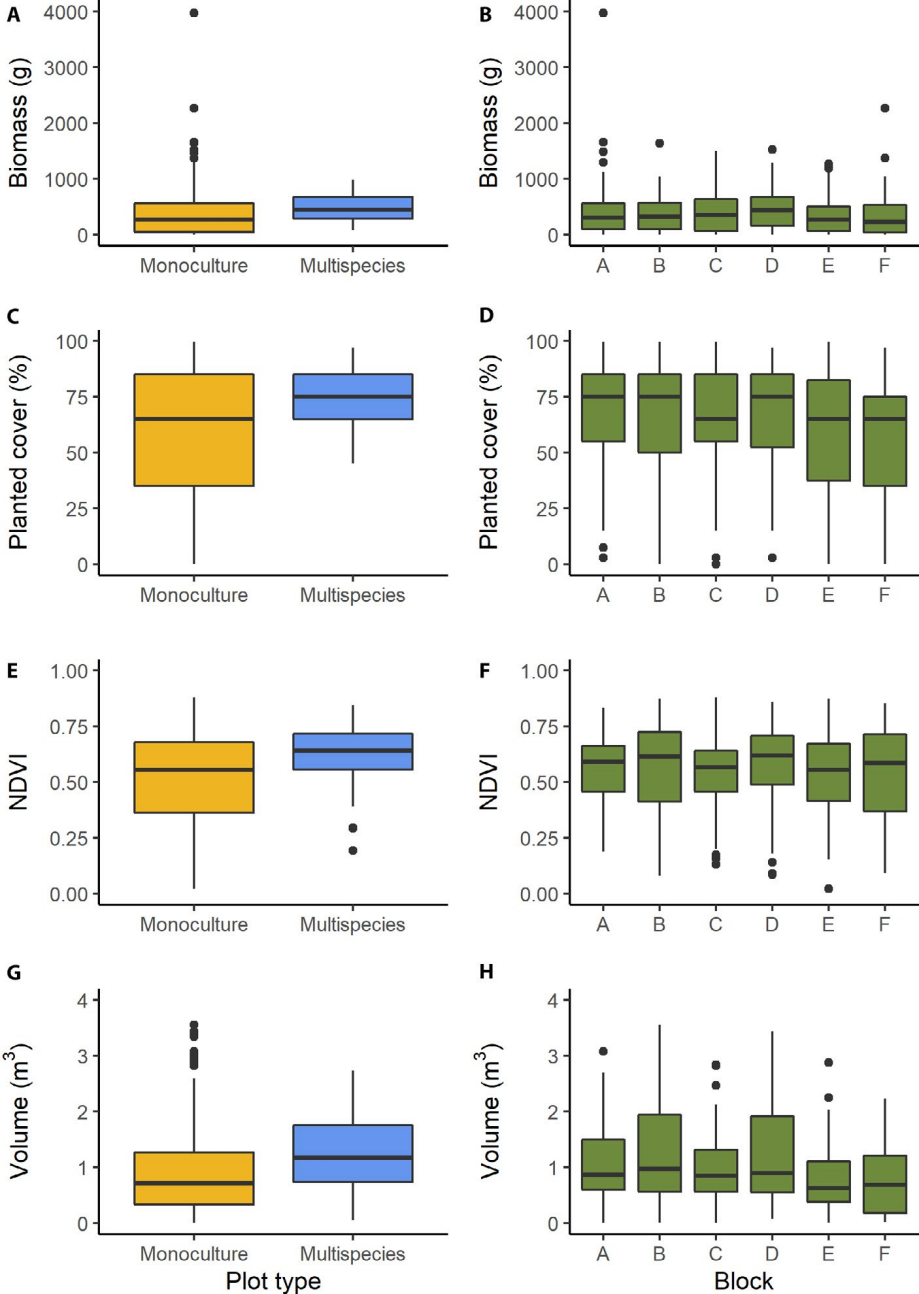
Boxplots of biomass (A, B), planted cover (C, D), NDVI (E, F), and volume (G, H), separated by plot type (A, C, E, G) and block (B, D, F, H).

The biomass of the monoculture plots ranged from 0 to 3977 g per 4‐m^2^ plot, with a mean of 399.0 g and a standard error of 29.1 g (Table 2, Fig. 3). There was no effect of soil blocking on the biomass (Kruskal–Wallis test, *k* = 6.15, *P* = 0.292). We did not analyze these patterns in the multispecies plots, because the multispecies plots that were measured incorrectly were concentrated in a few blocks, the removal of which might introduce bias from block conditions. We do, however, show summaries of correctly measured multispecies plots in Fig. 3. All biomass measurements for the monoculture and multispecies plots are listed in Supplements 4 and 5 (available on GitHub and Zenodo, see Data Availability statement), respectively.

**TABLE 2:** Summary statistics for the productivity proxies measured.

	All		Monocultures		Multispecies			
Productivity proxies	Mean	Min, max	Mean	Min, max	Mean	Min, max	Plot type *P* value	Block *P* value
Biomass	410.88	0.00, 3977.62	398.97	0.00, 3977.62	479.62	78.80, 981.00	0.0016	0.4001
Planted cover	62.81	0.00, 99.50	62.77	0.00, 99.50	73.98	45.00, 97.00	0.003	0.2748
Total cover	74.14	0.00, 100.00	74	0.00, 100.00	83.68	47.50, 100.00	0.143	0.0026
NDVI	0.65	0.08, 0.88	0.65	0.08, 0.88	0.72	0.34, 0.85	0.0025	0.026
GNDVI	0.66	0.39, 0.82	0.67	0.39, 0.82	0.7	0.49, 0.79	0.0015	0.0037
GDVI^2^	0.85	0.15, 0.99	0.86	0.15, 0.99	0.92	0.53, 0.98	0.0086	0.0187
VOL	0.96	−0.31, 3.55	0.87	−0.31, 3.55	1.29	0.06, 2.73	0.0000	0.0076

Note

GDVI^2^ = generalized difference vegetation index; GNDVI = green normalized difference vegetation index; NDVI = normalized difference vegetation index; VOL = vegetation volume.

### Vegetation cover

The planted cover ranged from 0% to 99.5%, with a mean of 62.8% (Table 2, Fig. 3). The planted cover was significantly higher in the multispecies plots (74.0% ± 1.48% [SEM]) than the monoculture plots (62.8% ± 1.87%; two‐sided Mann–Whitney test: *w* = 11,005, *P* = 0.003). There was no effect of the block type on the planted cover (Kruskal–Wallis test, *k* = 6.34, *P* = 0.275).

The total cover ranged from 0% to 100%, with a mean of 74.1% and standard error of 1.4%. There was no effect of diversity on the total cover (μ_Monoculture_ = 74.0 ± 1.8, μ_Multispecies_ = 83.7 ± 1.1; two‐sided Mann–Whitney test: *w* = 10,176, *P* = 0.143), but there was a significant block effect (Kruskal–Wallis test, *k* = 18.33, *P* = 0.003).

### Vegetation indices

The 598 photo sets were used to build multispectral orthomosaics at a resolution of 1.15 cm/pix (Fig. 1). A 2‐m scale bar measured 1.99 m in the orthomosaic, indicating that the scaling of the orthomosaic had a precision of >99%. The mean GDVI^2^ (0.85 ± 0.01) was higher than NDVI (0.65 ± 0.01) and GNDVI (0.66 ± 0.01; Table 2; Kruskal–Wallis test: *k* = 355.78, *P* < 0.0001). Furthermore, NDVI, GNDVI, and GDVI^2^ were all significantly higher in the multispecies plots (μ_NDVI_ = 0.72 ± 0.01, μ_GNDVI_ = 0.70 ± 0.01, μ_GDVI2_ = 0.92 ± 0.01) than in the monoculture plots (μ_NDVI_ = 0.65 ± 0.01, μ_GNDVI_ = 0.67 ± 0.01, μ_GDVI2_ = 0.86 ± 0.01; two‐sided Mann–Whitney test, NDVI: *w* = 11,279, *P* = 0.003; GNDVI: *w* = 11,379, *P* = 0.002; GDVI^2^: *w* = 10,999, *P* = 0.009, Fig. 3) and varied among blocks (Kruskal–Wallis test, NDVI: *k* = 12.73, *P* = 0.026; GNDVI: *k* = 17.47, *P* = 0.004; GDVI^2^: *k* = 13.55, *P* = 0.019).

### Volume

The mean volume across all plots was 0.96 m^3^ and ranged from –0.31 m^3^ to 3.55 m^3^. The 17 negative volume estimates suggest there was little vegetation within those plots and that the ground may slope. The volume was higher in multispecies plots (1.29 ± 0.08 m^3^) than in monoculture plots (0.87 ± 0.05 m^3^; Table 2; Kruskal–Wallis test: *w* = 12,705, *P* < 0.0001) and varied among blocks (two‐sided Mann–Whitney test, *k* = 15.74, *P* = 0.008).

### Evaluation of biomass models

We evaluated models predicting biomass with combinations of covariates from three groups (a VI or single band, total or planted cover, volume), for a total of 89 models with between one and three covariates each. Including block as a random effect resulted in negligible improvement in model likelihood and consequently the AIC increased (poorer model fit) for all models (available as Supplements 6–8 on GitHub and Zenodo, see Data Availability statement). We therefore only discuss models without block as a random effect. Moreover, the rank order of models based on AIC using plots with and without flowers was similar to that of models using only plots without flowers (available as Supplement 9 on GitHub and Zenodo, see Data Availability statement), so we do not discuss the results of the analyses conducted solely on plots without flowers. Models predicting the biomass of monoculture (Table 3 and Supplement 10 [available on GitHub and Zenodo, see Data Availability statement]) and multispecies plots (Table 4 and Supplement 11 [available on GitHub and Zenodo, see Data Availability statement]) separately produced very different results. Because of the difference in the number of monoculture plots (*n* = 254) and multispecies plots (*n* = 72) used here, the models evaluating all plot types together (see Supplement 12 on GitHub and Zenodo, see Data Availability statement) were dominated by the monoculture plots, and were almost identical to the models of monoculture plots alone. We therefore report the results for monoculture and multispecies plots separately.

**TABLE 3:** Models of biomass in monoculture plots. Only models using planted cover and those using uncorrected VIs are shown, as well as models mentioned in the text. See Supplement 10 (available on GitHub and Zenodo, see Data Availability statement) for all models.

VI used	Cover used	VI	Cover	Volume	*R* ^2^	AIC	∆AIC
GDVI^2^	Planted	−0.14, *P* = 0.0351	0.49, *P* < 0.0001	0.36, *P* < 0.0001	0.461	591.37	0
NDVI	Planted	−0.11, *P* = 0.0997	0.47, *P* < 0.0001	0.37, *P* < 0.0001	0.457	593.14	1.77
GNDVI	Planted	−0.11, *P* = 0.1502	0.47, *P* < 0.0001	0.37, *P* < 0.0001	0.456	593.79	2.42
—	Planted	—	0.43, *P* < 0.0001	0.33, *P* < 0.0001	0.451	593.9	2.53
REG	Planted	−0.09, *P* = 0.2236	0.46, *P* < 0.0001	0.37, *P* < 0.0001	0.454	594.39	3.02
NIR	Planted	−0.09, *P* = 0.2552	0.46, *P* < 0.0001	0.38, *P* < 0.0001	0.454	594.58	3.21
RED	Planted	0.05, *P* = 0.4077	0.44, *P* < 0.0001	0.34, *P* < 0.0001	0.452	595.2	3.83
GRE	Planted	−0.02, *P* = 0.6753	0.43, *P* < 0.0001	0.33, *P* < 0.0001	0.451	595.72	4.35
NIR	Planted	0.15, *P* = 0.0257	0.53, *P* < 0.0001	—	0.395	617.94	26.57
REG	Planted	0.11, *P* = 0.0985	0.56, *P* < 0.0001	—	0.39	620.22	28.85
—	Planted	—	0.62, *P* < 0.0001	—	0.383	620.99	29.62
GNDVI	Planted	0.07, *P* = 0.3183	0.58, *P* < 0.0001	—	0.385	621.98	30.61
GDVI^2^	Planted	−0.04, *P* = 0.5205	0.65, *P* < 0.0001	—	0.384	622.57	31.2
NDVI	Planted	0.03, *P* = 0.6771	0.61, *P* < 0.0001	—	0.383	622.81	31.44
GRE	Planted	0.01, *P* = 0.7781	0.62, *P* < 0.0001	—	0.383	622.91	31.54
RED	Planted	0.01, *P* = 0.8138	0.63, *P* < 0.0001	—	0.383	622.93	31.56
GDVI^2^	Total	−0.16, *P* = 0.0626	0.33, *P* = 1e‐04	0.50, *P* < 0.0001	0.384	635.55	44.18
GNDVI	—	0.14, *P* = 0.0443	—	0.50, *P* < 0.0001	0.353	645.99	54.62
NIR	—	0.13, *P* = 0.0880	—	0.49, *P* < 0.0001	0.35	647.14	55.77
REG	—	0.11, *P* = 0.1202	—	0.52, *P* < 0.0001	0.349	647.64	56.27
NDVI	—	0.10, *P* = 0.1272	—	0.54, *P* < 0.0001	0.349	647.73	56.36
—	—	—	—	0.60, *P* < 0.0001	0.343	648.09	56.72
GDVI^2^	—	0.08, *P* = 0.1890	—	0.55, *P* < 0.0001	0.347	648.34	56.97
RED	—	−0.06, *P* = 0.2922	—	0.58, *P* < 0.0001	0.346	648.96	57.59
GRE	—	−0.04, *P* = 0.4989	—	0.60, *P* < 0.0001	0.344	649.63	58.26
NIR	—	0.50, *P* < 0.0001	—	—	0.248	682.11	90.74
REG	—	0.46, *P* < 0.0001	—	—	0.216	692.91	101.54
GNDVI	—	0.46, *P* < 0.0001	—	—	0.215	693.21	101.84
NDVI	—	0.42, *P* < 0.0001	—	—	0.173	706.41	115.04
GDVI^2^	—	0.37, *P* < 0.0001	—	—	0.135	717.85	126.48
RED	—	−0.24, *P* = 1e‐04	—	—	0.058	739.42	148.05
GRE	—	0.03, *P* = 0.6113	—	—	0.001	754.38	163.01

Note

— = no covariate was used; AIC = Akaike’s information criterion; ∆AIC = the difference between the AIC of the best model evaluated for that test and the model being reported; GDVI^2^ = generalized difference vegetation index; GNDVI = green normalized difference vegetation index; GRE = green; NDVI = normalized difference vegetation index; NIR = near infrared; REG = red edge; VI = vegetation index.

**TABLE 4:** Models of biomass in multispecies plots. Only models using planted cover and those using uncorrected VIs are shown. See Supplement 11 (available on GitHub and Zenodo, see Data Availability statement) for all models.

VI used	Cover used	VI	Cover	Volume	*R* ^2^	AIC	∆AIC
REG	—	−0.26, *P* = 0.1203	—	0.67, *P* < 0.0001	0.498	46.52	0
—	—	—	—	0.55, *P* < 0.0001	0.468	47.14	0.62
GRE	—	−0.12, *P* = 0.2221	—	0.56, *P* < 0.0001	0.487	47.52	1
NIR	—	−0.18, *P* = 0.2333	—	0.64, *P* < 0.0001	0.486	47.6	1.08
REG	Total	−0.30, *P* = 0.1014	0.11, *P* = 0.5656	0.66, *P* < 0.0001	0.502	48.15	1.63
RED	—	−0.12, *P* = 0.4167	—	0.52, *P* < 0.0001	0.476	48.43	1.91
REG	Planted	−0.27, *P* = 0.1239	0.03, *P* = 0.8134	0.67, *P* < 0.0001	0.499	48.46	1.94
GDVI^2^	—	−0.09, *P* = 0.6474	—	0.57, *P* < 0.0001	0.47	48.92	2.4
—	Planted	—	−0.03, *P* = 0.8335	0.55, *P* < 0.0001	0.468	49.09	2.57
NDVI	—	−0.02, *P* = 0.9047	—	0.55, *P* < 0.0001	0.468	49.13	2.61
GNDVI	—	−0.02, *P* = 0.9171	—	0.56, *P* = 1e‐04	0.468	49.13	2.61
GRE	Planted	−0.12, *P* = 0.2343	0.01, *P* = 0.9497	0.55, *P* < 0.0001	0.487	49.52	3
NIR	Planted	−0.19, *P* = 0.2429	0.02, *P* = 0.8837	0.64, *P* < 0.0001	0.486	49.58	3.06
RED	Planted	−0.13, *P* = 0.4029	−0.04, *P* = 0.7525	0.54, *P* < 0.0001	0.477	50.32	3.8
GDVI^2^	Planted	−0.08, *P* = 0.6652	−0.02, *P* = 0.8714	0.58, *P* < 0.0001	0.471	50.89	4.37
NDVI	Planted	−0.01, *P* = 0.9333	−0.03, *P* = 0.8493	0.56, *P* < 0.0001	0.468	51.09	4.57
GNDVI	Planted	−0.02, *P* = 0.9311	−0.03, *P* = 0.8415	0.56, *P* = 2e‐04	0.468	51.09	4.57
GNDVI	—	0.56, *P* = 0.0010	—	—	0.229	63.45	16.93
GNDVI	Planted	0.52, *P* = 0.0043	0.12, *P* = 0.4476	—	0.24	64.82	18.3
NDVI	—	0.45, *P* = 0.0068	—	—	0.162	67.1	20.58
NDVI	Planted	0.39, *P* = 0.0264	0.14, *P* = 0.3947	—	0.177	68.31	21.79
NIR	—	0.36, *P* = 0.0159	—	—	0.131	68.7	22.18
REG	—	0.36, *P* = 0.0313	—	—	0.106	69.96	23.44
NIR	Planted	0.30, *P* = 0.0723	0.13, p = 0.4762	—	0.142	70.15	23.63
GDVI^2^	—	0.44, *P* = 0.0375	—	—	0.099	70.28	23.76
GDVI^2^	Planted	0.36, *P* = 0.0984	0.20, *P* = 0.2262	—	0.131	70.69	24.17
REG	Planted	0.28, *P* = 0.1289	0.15, *P* = 0.3901	—	0.122	71.16	24.64
RED	Planted	−0.29, *P* = 0.1315	0.23, *P* = 0.1659	—	0.121	71.19	24.67
RED	—	−0.35, *P* = 0.0655	—	—	0.078	71.27	24.75
—	Planted	—	0.28, *P* = 0.0818	—	0.07	71.66	25.14
GRE	Planted	−0.12, *P* = 0.3611	0.32, *P* = 0.0578	—	0.089	72.75	26.23
GRE	—	−0.06, *P* = 0.6590	—	—	0.005	74.66	28.14

Note

— = no covariate was used; AIC = Akaike’s information criterion; ∆AIC = the difference between the AIC of the best model evaluated for that test and the model being reported; GDVI^2^ = generalized difference vegetation index; GNDVI = green normalized difference vegetation index; GRE = green; NDVI = normalized difference vegetation index; NIR = near infrared; REG = red edge; VI = vegetation index.

Planted cover (30/89 models; cumulative AICw = 0.999) and volume (45/89 models; cumulative AICw = 0.999) held by far the most predictive power, followed distantly by GDVI^2^ (6/89 models; cumulative AICw = 0.252). The cumulative AICw of all other predictors was less than 0.104. The best model for predicting biomass in monoculture plots used GDVI^2^, volume, and planted cover as predictors, and accounts for 46% of the variation in biomass. All models incorporating planted cover and volume account for approximately 45% of the variation in biomass and have ΔAIC values <4.6. All models without both planted cover and volume have ΔAIC > 26.57 and *R* ^2^ < 0.42. The best model for predicting monoculture plots using only metrics from remote sensing imagery is similar to the best overall, but uses total cover, estimated by a visual inspection of aerial imagery, instead of planted cover, estimated by a visual inspection at ground level (*R* ^2^ = 0.38, *P* < 2.2 × 10^−16^).

The most important predictors of multispecies plots were volume (45/89 models, AICw = 0.999), total cover (30/89 models, AICw = 0.217), and planted cover (30/89 models, AICw = 0.215). Models incorporating volume have ΔAIC < 4.62 and account for at least 47% of the variance in biomass, whereas models that do not include volume have ΔAIC > 16.9 and account for less than 23% of the variance in biomass. The best model for predicting multispecies plots using only remotely sensed variables uses the red edge band, total cover, and volume (*R* ^2^ = 0.50).

Across all models, the residuals were nonlinear and heteroskedastic, suggesting the covariates we used did not account for all the variation in the response.

### Cover models

We evaluated models predicting cover with combinations of covariates produced by selecting one variable from each of two groups (VI or single band and volume), for a total of 15 models with one or two covariates each. Models including all plots were dominated by monocultures (see Supplement 13 on GitHub and Zenodo, see Data Availability statement), and models predicting including and excluding flowers were very similar (see Supplement 14 on GitHub and Zenodo, see Data Availability statement). Similar to the models predicting biomass, including block as a random effect increased the AIC in all models (see Supplements 15–17 on GitHub and Zenodo, see Data Availability statement).

The best model for predicting cover in monoculture plots uses GDVI^2^ and volume and accounts for 67% of the variance in biomass. The AIC and *R* ^2^ scores are similar for models using only a VI and models using the same VI with volume (ΔAIC < 2), except in the case of GDVI^2^, for which the model using volume is better (ΔAIC = 10.3). The ΔAIC is >105.99 for the models using single bands instead of VIs (Table 5). Volume (8/15 models) and GDVI^2^ (2/15 models) are the most important covariates for predicting cover in monoculture plots (cumulative AICw = 0.999 and 0.994, respectively), with the AICw of all other covariates less than 1.5 × 10^−6^.

**TABLE 5:** Models of total cover in monoculture plots.

VI used	VI	Volume	*R* ^2^	AIC	∆AIC
GDVI^2^	0.72, *P* < 0.0001	0.16, *P* = 5e‐04	0.661	483.05	0
GDVI^2^	0.80, *P* < 0.0001	—	0.644	493.35	10.3
NDVI	0.73, *P* < 0.0001	0.10, *P* = 0.0497	0.622	510.61	27.56
NDVI	0.79, *P* < 0.0001	—	0.616	512.52	29.47
GNDVI	0.78, *P* < 0.0001	—	0.607	518.76	35.71
GNDVI	0.76, *P* < 0.0001	0.04, *P* = 0.4758	0.607	520.24	37.19
NIR	0.69, *P* < 0.0001	—	0.481	589.04	105.99
NIR	0.67, *P* < 0.0001	0.03, *P* = 0.6723	0.481	590.86	107.81
REG	0.58, *P* < 0.0001	0.13, *P* = 0.0481	0.463	599.7	116.65
REG	0.67, *P* < 0.0001	—	0.455	601.67	118.62
RED	−0.43, *P* < 0.0001	0.40, *P* < 0.0001	0.45	605.63	122.58
RED	−0.56, *P* < 0.0001	—	0.31	661.45	178.4
—	—	0.55, *P* < 0.0001	0.287	669.88	186.83
GRE	−0.04, *P* = 0.4078	0.55, *P* < 0.0001	0.289	671.18	188.13
GRE	0.02, *P* = 0.7886	—	0	755.59	272.54

Note

— = no covariate was used; AIC = Akaike’s information criterion; ∆AIC = the difference between the AIC of the best model evaluated for that test and the model being reported; GDVI^2^ = generalized difference vegetation index; GNDVI = green normalized difference vegetation index; GRE = green; NDVI = normalized difference vegetation index; NIR = near infrared; REG = red edge; VI = vegetation index.

All multispecies‐plot models (Table 6) have lower *R* ^2^ values than models predicting monoculture cover. The best model for multispecies plots uses only GNDVI and accounts for 29% of the variation in cover. Volume (8/15 models), GNDVI (2/15 models), and red edge (2/15 models) are the most important predictors of cover in multispecies plots (AICw = 0.384, 0.358, and 0.232, respectively).

**TABLE 6:** Models of total cover in multispecies plots.

VI used	VI	Volume	*R* ^2^	AIC	∆AIC
GNDVI	0.46, *P* = 1e‐04	—	0.293	29.89	0
REG	0.41, *P* = 3e‐04	—	0.275	31.01	1.12
GNDVI	0.42, *P* = 0.0106	0.03, *P* = 0.7565	0.295	31.79	1.9
NIR	0.36, *P* = 4e‐04	—	0.258	32.05	2.16
NDVI	0.41, *P* = 4e‐04	—	0.257	32.07	2.18
REG	0.35, *P* = 0.0158	0.07, *P* = 0.5169	0.282	32.55	2.66
NDVI	0.32, *P* = 0.0198	0.10, *P* = 0.3165	0.275	32.98	3.09
RED	−0.28, *P* = 0.0256	0.18, *P* = 0.0250	0.267	33.47	3.58
NIR	0.30, *P* = 0.0259	0.07, *P* = 0.4732	0.267	33.49	3.6
GDVI^2^	0.28, *P* = 0.0892	0.15, *P* = 0.1058	0.228	35.74	5.85
GDVI^2^	0.42, *P* = 0.0045	—	0.177	36.58	6.69
—	—	0.24, *P* = 0.0052	0.171	36.88	6.99
RED	−0.36, *P* = 0.0053	—	0.171	36.92	7.03
GRE	0.01, *P* = 0.9353	0.23, *P* = 0.0061	0.172	38.87	8.98
GRE	0.03, *P* = 0.7293	—	0.003	45.03	15.14

Note

— = no covariate was used; AIC = Akaike’s information criterion; ∆AIC = the difference between the AIC of the best model evaluated for that test and the model being reported; GDVI^2^ = generalized difference vegetation index; GNDVI = green normalized difference vegetation index; GRE = green; NDVI = normalized difference vegetation index; NIR = near infrared; REG = red edge; VI = vegetation index.

### Phylogenetic signal of functional traits correlated with biomass and NDVI

**FIGURE 4:**
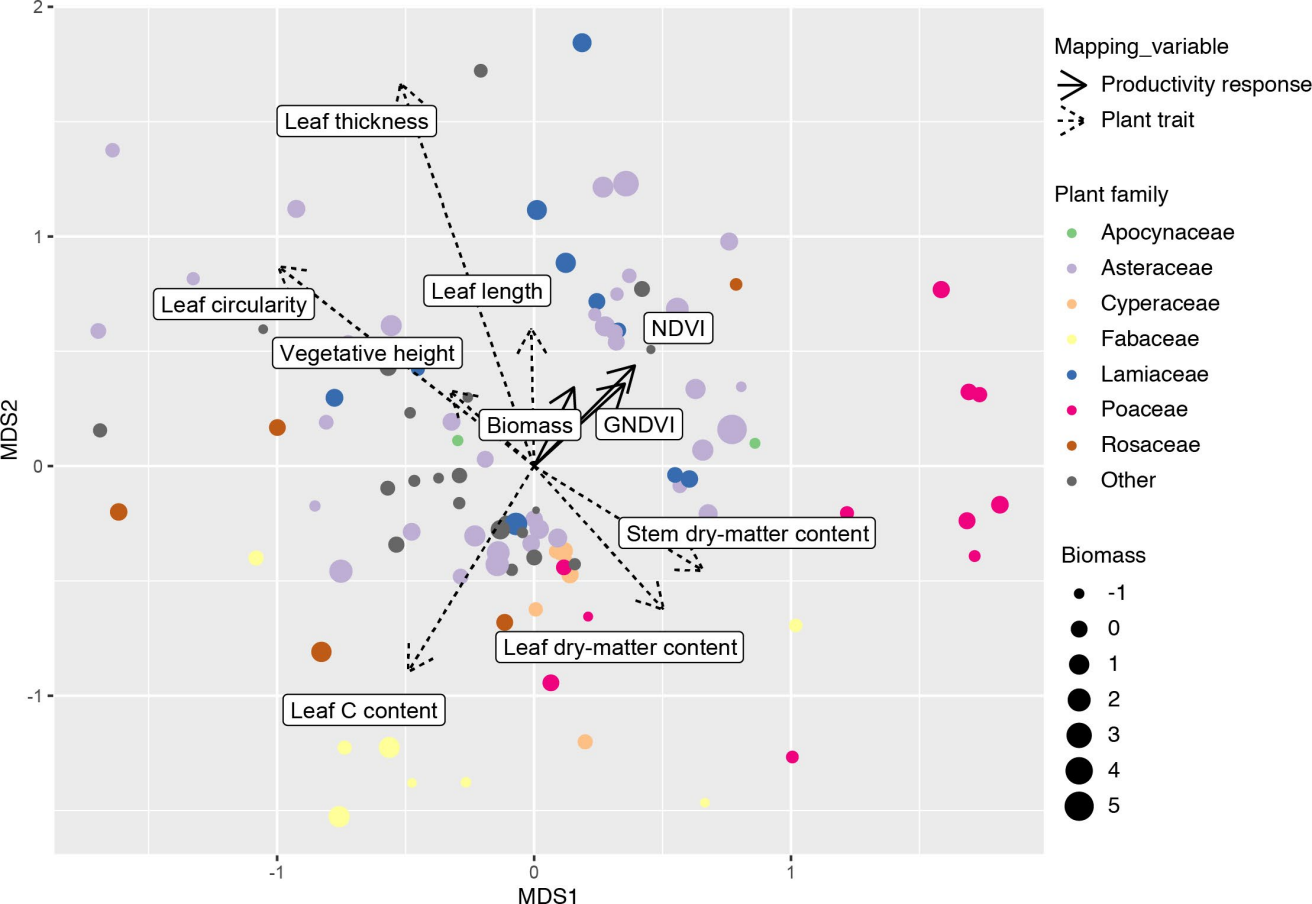
Non‐metric multidimensional scaling ordination of all 127 species, given their functional traits. Point size shows biomass centered and scaled to have a mean of 0 and standard deviation of 1.

**FIGURE 5:**
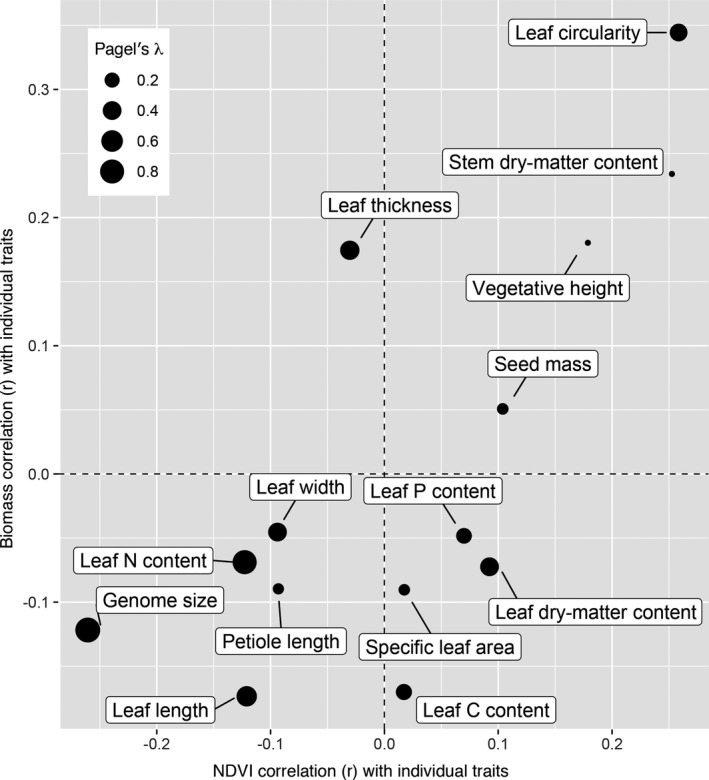
Correlation of biomass and NDVI with individual traits. Size of dots represent phylogenetic heritability, as estimated using Pagel’s *λ*. The traits with greatest heritability (lower left; i.e., leaf nitrogen [N] content and genome size) are weakly correlated with biomass and NDVI, whereas traits with a high correlation have low heritability (upper right; i.e., stem dry‐matter content, vegetative height).

Traits exhibit high variation in phylogenetic heritability (see Supplement 18 on GitHub and Zenodo, see Data Availability statement), but overall the multivariate trait space is moderately predicted by phylogeny, with plants largely intermixed in trait space by family, although the Fabaceae and Poaceae stand out as distinct (Fig. 4). Stem dry‐matter content and vegetative height, two of the traits most strongly correlated with productivity, show low phylogenetic heritability (stem dry‐matter content: *λ* < 0.00001, *K* = 0.00263; vegetative height: *λ* < 0.00001, *K* = 0.00651) (Fig. 5, Supplement 18 [available on GitHub and Zenodo, see Data Availability statement]). Leaf thickness and circularity are also strongly correlated with biomass and have moderate estimates of phylogenetic heritability (*λ* = 0.43687 and 0.33924, respectively). Seed mass is weakly positively correlated with productivity and has moderate phylogenetic heritability (*λ* = 0.0771). The three traits with particularly strong phylogenetic heritability (genome size, *λ* = 0.88188; leaf nitrogen content, *λ* = 0.80715; leaf length, *λ* = 0.5112; and leaf width, *λ* = 0.40038) were weakly correlated with biomass and NDVI (Fig. 5). All measures of Blomberg’s *K* were significantly less than 1, suggesting that the variance for each trait measured is relatively high within the sampled clades vs. among clades. Genome size, leaf nitrogen content, and specific leaf area had the highest measures (*K* = 0.02821, 0.01307, and 0.01013, respectively).

The correlation between the pairwise VI dissimilarity and phylogenetic distance was not statistically significant (see Supplement 19 on GitHub and Zenodo, see Data Availability statement), but biomass was significantly correlated with phylogeny using the same Mantel tests (*r* = 0.4476, *P* < 0.001). Our pairwise species‐level VIs were also not significantly correlated with the pairwise VIs described by Schweiger et al. (2018) (*r* = 0.0089, *P* = 0.468), likely because our VIs were collected at the plot level instead of the leaf level and thus less closely tied to individual species.

## DISCUSSION

### Predictive ability depends on vegetation diversity

Our study demonstrates that the predictive ability of all remote sensing metrics varies depending on diversity, but indicates that the drone‐based quantification of vegetation volume is a useful estimator in both monoculture and multispecies plots, explaining 34% and 47% of the variation in biomass, respectively. Of the predictors we tested for biomass, we found that volume is the most important for multispecies plots (cumulative AICw = 0.999) and second‐most important (after planted cover) for monoculture plots (cumulative AICw = 0.999). Only one combination of covariates (volume and red edge) estimates biomass in multispecies plots better than volume alone (*R* ^2^ = 0.50, ΔAIC = 0.62). Conversely, volume is less effective at predicting biomass in monoculture plots than 58/89 combinations of covariates based on ΔAIC (volume and total or planted cover with or without any VI or single band; planted cover with or without any VI or single band; planted cover alone; volume and corrected or uncorrected NDVI, GNDVI, red edge, or near‐infrared, or corrected green or red), which explain up to 46% of the variation in biomass (see Supplement 10 on GitHub and Zenodo, see Data Availability statement). This finding is particularly interesting because the variance in biomass among monoculture plots is higher than the variance among multispecies plots (Fig. 3). This is what we would expect, as the monoculture plots contain the extremes of growth form whereas multispecies plots are weighted averages of these extremes; consequently, it is hard to see how the variance of mixture plot variance could not be less than the variance of the monoculture plot variance.

Volume may explain more of the variance in multispecies biomass than monoculture biomass if high‐diversity plots have greater vertical niche partitioning and thus show a tighter correlation between volume and biomass due to a more complete filling of the volume estimated by photogrammetry. Vertical niche partitioning may result in more efficient space filling in the multispecies plots: species with different growth forms may exploit vertical layers more efficiently, mirroring canopy packing in diverse forests (Jucker et al., 2015). In monocultures, individuals of the same species are more likely to occupy the same level, leading to empty space under the highest vegetation layer. Because volume is estimated from the outermost layer of vegetation only, empty space will result in lower measured biomass but will have no effect on estimated volume. Therefore, monocultures will likely have a looser relationship between biomass and volume. Because of the limitations of our sampling that make it impossible to tease apart complementarity from selection effects in the multispecies plots (see discussion above), we cannot directly test the hypothesis here. To further investigate this relationship, LiDAR metrics that identify the density of vegetation at different heights could be useful, as would future analyses in which monoculture biomass and multispecies plot biomass were measured in the same way.

Surprisingly, although NDVI is one of the most commonly used VIs (Pettorelli et al., 2005), it was not the best VI predictor of biomass or vegetation cover in either plot type in our experimental prairie. Based on AICw, GDVI^2^ was more important in predicting both biomass and cover (cumulative AICw = 0.252 and 0.999, respectively) than NDVI (cumulative AICw = 0.104 and 1.43 × 10^−6^, respectively) in monoculture plots. In multispecies plots, red edge was the most important spectral predictor of biomass (cumulative AICw = 0.160) and GNDVI was the most important spectral predictor of cover (cumulative AICw = 0.385); by contrast, NDVI was the least important spectral predictor of biomass (cumulative AICw = 0.042) and the third‐most important spectral predictor of cover (cumulative AICw = 0.152). The difference in the best spectral predictors of biomass is likely related to the difference in vegetation density between monoculture and multispecies plots: GDVI^2^ is more dynamic for less‐dense vegetation (Wu, 2014), whereas red edge is a good indicator of biomass at higher vegetation densities (Cao et al., 2016). Similarly, GDVI^2^ and GNDVI likely predict cover in monocultures and multispecies plots best because they are more sensitive to variation at low and high vegetation densities, respectively (Gitelson et al., 1996; Wu, 2014). We expect that the patterns we describe here will be generalizable to other grassland community types that are similar in diversity, density, and stratification.

### Prairie productivity is driven by phylogenetically labile traits

The traits most strongly correlated with productivity in our study are also traits with low phylogenetic heritability (Fig. 5). Although traits evolving without constraint or selection are expected to track phylogeny (Felsenstein, 1985), natural selection dampens the effect of phylogenetic heritage on trait variation if it is uniform across taxa (e.g., in stabilizing selection) (Hansen, 1997), and even more dramatically when there is convergence between clades in ecologically significant traits (e.g., Cavender‐Bares et al., 2004). Our results suggest that traits responsible for productivity—estimated in our study as biomass and VIs—are more variable within clades than between clades, either due to convergence, stabilizing selection, or pleiotropy on other selected traits. This is not true of all traits; in multivariate space, some plant families segregated based on functional traits (Fig. 4). This is expected if traits in general tend to track phylogeny; while individual traits diverge even under neutral expectations, even a wide range of traits that all fail to track phylogeny closely may track it in aggregate (Givnish and Sytsma, 1997; Cadotte et al., 2017). The mint family (Lamiaceae) and sunflower family (Asteraceae) are particular exceptions in our study as they cover a relatively broad range of trait space, suggesting that trait variance within these lineages may be driving the observed patterns.

These results suggest that additional unmeasured traits associated with phylogeny may drive aboveground productivity. Alternatively, we may be observing a biodiversity effect (Duffy et al., 2017) that correlates with phylogeny, even if individual traits do not. Trait correlations with both productivity and phylogeny may be more complex than our metrics suggest; leaf dry matter content, for example, has been shown to be correlated with productivity (Smart et al., 2017), but is inconsistently correlated with productivity metrics in the current study and exhibits moderate phylogenetic conservatism (Fig. 5). Furthermore, we also assessed whether measures of productivity were correlated with traits that influence productivity, such as leaf carbon content. Although NDVI has been used as a predictive tool for measures of biomass and leaf nitrogen content (Cabrera‐Bosquet et al., 2011), we did not find such a correlation with leaf carbon content, likely because the spectra involved in distinguishing the levels of carbon are different from those involved in detecting nitrogen levels.

### Application of high‐resolution imagery in ecological studies

Remote sensing metrics are often used to detect productivity (Wang et al., 2004), as well as species richness and diversity (Gould, 2000); however, few studies examine these three features together (Wang et al., 2016). Here, we find that these three features are interrelated: vegetation diversity affects the relationship between remote sensing metrics and productivity. Specifically, these results suggest that when using metrics derived from imagery to estimate productivity, diversity needs to be considered. We focus on only two levels of diversity (monoculture and multispecies), but future studies using more levels could examine this relationship in more detail.

Our finding that volume is more useful than NDVI in estimating biomass has two important implications: (1) a traditional camera can capture adequate RGB images for volume reconstruction, reducing costs compared to techniques requiring a multispectral sensor; and (2) LiDAR approaches may prove even more accurate, given the informative vegetation structure data. The VIs, particularly NDVI, have been the focus of most previous biomass estimations (Das and Singh, 2012; Goswami et al., 2015), but our results support recent research suggesting that high‐resolution volume data are also a good estimate of biomass (Wallace et al., 2017).

We demonstrate the use of high‐resolution remote sensing imagery to address an ecological question. We find that volume is a good predictor of biomass regardless of diversity. Because structural metrics such as volume are the least expensive remote sensing proxy to obtain, this technique could be used much more widely than those requiring a multispectral sensor. Finally, we find that the relationship between remote sensing proxies of productivity depends on the diversity of the vegetation. The variation in this relationship is likely due to differences in vegetation structure and warrants further investigation.

## AUTHOR CONTRIBUTIONS

C.L.S., A.L.H., M.C.G., and B.C.S. designed the experiment. C.L.S., M.C.G., A.T., A.L.H., and B.C.S. conducted the experiment and collected data. C.L.S., N.K., and A.L.H. analyzed the data. C.L.S., A.L.H., N.K., C.H.C., A.T., and B.C.S. wrote and revised the manuscript. All authors approved the final version.

## Supporting information


**APPENDIX S1.** Detailed methods used in this study.

**APPENDIX S2.** Parameters used for photo processing and orthomosaic production.

## Data Availability

Scripts and data used in these analyses, as well as all supplements referenced in this article, are available on GitHub (https://github.com/lanescher/prairie‐remote‐sensing‐2020) and on Zenodo (https://doi.org/10.5281/zenodo.3981500; Scher et al., 2020).
