# Supplementary material for: Application of remote sensing technology to estimate productivity and assess phylogenetic heritability
Source: Appl Plant Sci. 2020 Nov 29;8(11):e11401. doi: 10.1002/aps3.11401 (PMC7705335; doi:10.1002/aps3.11401)
Supplement: Supplementary file 1 — APPENDIX S1. Detailed methods used in this study. [file APS3-8-e11401-s001.docx]

**Appendix S1.** Detailed methods used in this study.

**Soils**

Two to three soil cores (2.5 cm wide) were collected from each plot with a footstep soil probe (Oakfield Apparatus, Oakfield, Wisconsin, USA). The A horizon was measured at the occurrence of a visible color change; if no change occurred, the depth was recorded as beyond the maximum depth sampled (40 cm). The areas of redoximorphic concentrations and depletions were estimated on the exposed profile face. A total of 17 plots per block were randomly selected and measured for additional characteristics: gravimetric soil moisture, loss on ignition soil organic matter, pH, electrical conductivity, wet aggregate stability, and phosphorus. The remaining plots were interpolated using kriging, where missing plots were estimated as the weighted average of surrounding plots. Kriging was conducted in the kriging package version 1.1 (Olmedo, 2014) for R (R Core Team, 2019), with pixels separated by 0.5 m and a lag of 10 pixels. For each plot, the resulting value used in the estimate was taken as the mean of the pixels that fall within 1 m of the center of each plot.

The soil cores were refrigerated at 3°C until processing, at which time they were sieved through a 6-mm screen. Soil subsamples were then weighed, dried for 24 h at 105°C, and reweighed to gravimetrically calculate the soil moisture (Topp et al., 2008). Loss on ignition was performed at 360°C for a minimum of three days to determine the soil organic matter (Nelson and Sommers, 1996). The stability of aggregates (1–2-mm size class) was measured by the oscillation of the sample through a height of 37 mm, 29 times per minute for 10 min in water (Angers et al., 2008). The oven-dry weight of water-stable aggregates per total oven-dry soil was expressed as a percentage (Scharenbroch and Catania, 2012).

Soil pH and electrical conductivity in dS cm^–1^ were measured in 1 : 1 (soil : deionized) water pastes using a multifunction meter (Orion 5-Star; Thermo Fisher Scientific, Waltham, Massachusetts, USA). Soil phosphorus was determined with the Bray extraction method and extracts were analyzed colorimetrically at 882 nm on a spectrophotometer (UV mini 1240; Shimadzu, Kyoto, Japan) (Olsen and Sommers, 1982).

**Drone flight**

We used an automated flight path designed with Litchi flight planning software (VC Technology Ltd., London, United Kingdom). To ensure both high overlap and high resolution, the drone traversed the prairie twice at 10 m above the ground and twice at 15 m above the ground, traveling at speeds of between 6.0 and 6.1 km h^–1^. A set of photos, each consisting of one RGB photo (16 Mpix) and four single-band images of red, green, near infrared, and red edge (1.2 Mpix), was captured every 4 s, for a total of 598 sets of five photos. All parts of the prairie were visible in at least nine photo sets, indicating that there was sufficient overlap to produce reliable orthomosaics. The orthomosaics were produced from the photos using Agisoft Photoscan (version 1.4.3 build 6529; Agisoft, St. Petersburg, Russia) with the parameters listed in Appendix S2.

**Cover correction factor**

The correction factor for each plot was calculated by subtracting the total cover from the planted cover. All pixels in each plot were classified as bare “soil” (normalized difference vegetation index [NDVI] < 0.1) or “vegetation” (NDVI > 0.1) based on the NDVI score. In plots with positive correction values (growth occurred between photo capture and biomass measurement), a portion of soil pixels equal to the correction factor was randomly chosen to be replaced with vegetation pixels. The values of these soil pixels were replaced with the values of a random vegetation pixel in the same plot. In plots with negative correction values (weeds present in aerial photos), vegetation pixels were replaced with soil pixels using the same method.

Total cover and planted cover were highly correlated (*r* = 0.73, *P* < 2.2 × 10^–16^). The correction factor ranged from 25 to –80, with a mean of –12.37.

**Trait data**

For species without published data, we collected samples opportunistically from sites within The Morton Arboretum, or from known populations at two sites in the vicinity of The Morton Arboretum (Chicago Botanic Garden, Glencoe, Illinois, USA, and Fermi National Accelerator Lab, Batavia, Illinois, USA). Up to 10 individuals were collected from multiple sites, for an average of seven individuals collected per species. For the measurement of morphological traits, we collected two mature leaves from each individual, specifically one basal and one cauline leaf if they differed markedly in shape or size. All leaves were kept separate throughout the measurement process. We targeted mature leaves largely free of herbivory and pathogen damage and we made notes if samples did not meet these requirements.

Any trait data missing from the data matrix were imputed using multivariate imputation by chained equations with the MICE package (van Buuren and Groothuis-Oudshoorn, 2011) for R. Our trait matrix with missing data was first converted to a dissimilarity matrix using Gower’s distance (Gower, 1971). We then used a predictive mean matching model for numeric data and logistic regression imputation for our binary data with two factor levels and polytomous regression imputation for unordered categorical data.

**LITERATURE CITED**

Angers, D. A., M. S. Bullock, and G. R. Mehuys. 2008. Aggregate stability to water. *In* M. R. Carter and E. G. Gregorich [eds.], Soil sampling and methods of analysis, 811–820. Canadian Society of Soil Science, CRC Press, Boca Raton, Florida, USA.

Gower, J. C. 1971. A general coefficient of similarity and some of its properties. *Biometrics* 27: 857–871.

Nelson, D. W., and L. E. Sommers. 1996. Total carbon, organic carbon, and organic matter. *In* D. L. Sparks [ed.]. Methods of soil analysis. Part 3: Chemical Methods, 961–1010. Soil Science Society of America, Madison, Wisconsin, USA.

Olmedo, O. E. 2014. kriging: Ordinary Kriging. R package version 1.1. Website https://CRAN.R-project.org/package=kriging [accessed 13 October 2020].

Olsen, S. R., and L. E. Sommers. 1982. Phosphorus. *In* A. L. Page, R. H. Miller, and D. R. Keeney [eds.], Methods of soil analysis. Part 2. Chemical and microbiological properties, 403–430. American Society of Agronomy, Soil Science Society of America, Madison, Wisconsin, USA.

R Core Team. 2019. R: A language and environment for statistical computing. The R Foundation for Statistical Computing, Vienna, Austria. Website https://www.r-project.org/ [accessed 13 October 2020].

Scharenbroch, B. C., and M. Catania. 2012. Soil quality attributes as indicators of urban tree performance. *Arboriculture and Urban Forestry* 38(5): 214–228.

Topp, G. C., G. W. Parkin, and T. P. A. Ferre. 2008. Soil water content. *In* M. R. Carter and E. G. Gregorich [eds.], Soil sampling and methods of analysis, 939–962. CRC Press, Boca Raton, Florida, USA.

van Buuren, S., and K. Groothuis-Oudshoorn. 2011. MICE: Multivariate Imputation by Chained Equations in R. *Journal of Statistical Software* 45(3): v045i03.
