# Supplementary material for: Application of remote sensing technology to estimate productivity and assess phylogenetic heritability
Source: Appl Plant Sci. 2020 Nov 29;8(11):e11401. doi: 10.1002/aps3.11401 (PMC7705335; doi:10.1002/aps3.11401)
Supplement: Supplementary file 2 — APPENDIX S2. Parameters used for photo processing and orthomosaic production. [file APS3-8-e11401-s002.docx]

**APPENDIX S2**. Parameters used for photo processing and orthomosaic production.

| **Parameter** | **Setting** |
| --- | --- |
| Align photos | |
| Accuracy | Highest |
| Pre-selection | Generic, reference |
| Key point limit | 0 |
| Tie point limit | 0 |
| Adaptive camera model fitting | Yes |
| Build dense cloud | |
| Quality | Ultra high |
| Depth filtering | Mild |
| Reuse depth maps | No |
| Calculate point color | Yes |
| Build mesh | |
| Surface type | Arbitrary |
| Source data | Dense |
| Face count | High |
| Interpolation | Disabled |
| Calculate vertex colors | Yes |
